# Spontaneous circadian rhythms in a cold-adapted natural isolate of *Aureobasidium pullulans*

**DOI:** 10.1038/s41598-017-14085-6

**Published:** 2017-10-23

**Authors:** Diana L. Franco, Paulo Canessa, Nicolás Bellora, Sebastián Risau-Gusman, Consuelo Olivares-Yañez, Rodrigo Pérez-Lara, Diego Libkind, Luis F. Larrondo, Luciano Marpegan

**Affiliations:** 1 Instituto de Investigaciones en Biodiversidad y Medio Ambiente (INIBIOMA), Universidad Nacional del Comahue, CONICET, CRUB, San Carlos de Bariloche, Río Negro, Argentina; 2 Centro de Biotecnologia Vegetal, Facultad de Ciencias Biologicas, Universidad Andres Bello, Santiago, Chile; 3 Millennium Nucleus for Fungal Integrative and Synthetic Biology (MN-FISB), Santiago, Chile; 4 CONICET-Centro Atómico Bariloche, Bariloche, Rio Negro, Argentina; 5 Departamento de Genética Molecular y Microbiología, Facultad de Ciencias Biológicas, Pontificia Universidad Católica de Chile, Santiago, Chile; 6 Departamento de Ciencia y Tecnología, Universidad Nacional de Quilmes, Bernal, Buenos Aires Argentina; 7 Present Address: Departamento de Física Médica Centro Atómico Bariloche and Instituto Balseiro, CONICET, San Carlos de Bariloche, Río Negro, Argentina

## Abstract

Circadian systems enable organisms to synchronize their physiology to daily and seasonal environmental changes relying on endogenous pacemakers that oscillate with a period close to 24 h even in the absence of external timing cues. The oscillations are achieved by intracellular transcriptional/translational feedback loops thoroughly characterized for many organisms, but still little is known about the presence and characteristics of circadian clocks in fungi other than *Neurospora crassa*. We sought to characterize the circadian system of a natural isolate of *Aureobasidium pullulans*, a cold-adapted yeast bearing great biotechnological potential. *A*. *pullulans* formed daily concentric rings that were synchronized by light/dark cycles and were also formed in constant darkness with a period of 24.5 h. Moreover, these rhythms were temperature compensated, as evidenced by experiments conducted at temperatures as low as 10 °C. Finally, the expression of clock-essential genes, *frequency*, *white collar-*1, *white collar-2* and *vivid* was confirmed. In summary, our results indicate the existence of a functional circadian clock in *A*. *pullulans*, capable of sustaining rhythms at very low temperatures and, based on the presence of conserved clock-gene homologues, suggest a molecular and functional relationship to well-described circadian systems.

## Introduction

Circadian systems are ubiquitous, they are present in organisms ranging from bacteria to humans, including plants, insects and fungi^1^, enabling synchronization of key biochemical, cellular and physiological processes to cyclic environmental events (mostly to daily and seasonal variations). Their wide distribution combined with experimental data from Cyanobacteria^2^, *Drosophila* ^3^, *Arabidopsis* ^4^ and mammals^5^ strongly suggest an adaptive advantage provided by functional circadian clocks. The relevance of circadian systems is also evidenced by the extent of transcriptional control over gene expression, with the whole genome in cyanobacteria, nearly half of all genes in mouse^6^ and up to 40% of the *Neurospora crassa*’s transcriptome being rhythmically expressed^7,8^ (reviewed by Wijnen & Young^9^).

The formal properties defining circadian rhythms are the same in all organisms studied so far and provide a simple –yet powerful– diagnostic criteria for identifying an endogenous circadian clock^10^. Circadian rhythms have the ability to persist in constant conditions with a period close to 24 h, which is temperature compensated (maintained in a physiological range of temperatures). In addition, circadian rhythms can be entrained by external signals such as the daily light and temperature cycles. A simple model of circadian system includes three principal components: a central oscillator (molecular clock), input pathways that convey environmental information and synchronize the oscillator to the external world, and output pathways that allow it to modulate most cellular processes including gene expression and cellular metabolism, among others. The general mechanism of the molecular clock, highly conserved among species, is an autonomous oscillator that consists of a series of interlocked feedback loops with several proteins negatively modulating their own transcriptional activation, thereby producing an oscillation in their abundances. These molecular oscillations are not only regulated at the transcriptional level but also at the post-translational level, particularly by changes in the phosphorylation status of the negative elements controlling protein stability, activity, dimerization and subcellular localizations^11^.

Because of their ecological and economical properties fungi are critical for humans and the environment. They are a key source of biotechnologically relevant products worldwide, despite only a small fraction of them currently being utilized or investigated. Fungi constitute an excellent model to study diverse biological processes including circadian clocks, because large populations can be utilized, they have short duplicating times, can be easily manipulated and there are several genetic tools allowing the generation of mutants. In fact, circadian rhythms have been studied for over fifty years in the filamentous fungus *N*. *crassa* ^12^. The daily control of asexual development in this ascomycete, was found to be an easily scorable phenotype, making it a practical model organism for circadian research^12^. The study of the core molecular oscillator in *N*. *crassa* revealed that the transcription factor/photoreceptor White Collar-1 (WC-1) associates with its partner White-Collar-2 (WC-2) forming the White Collar Complex (WCC), a positive element that activates the expression of the negative element: the *frequency* (*frq*) gene. FRQ protein is then synthetized; it associates with FRQ-interacting helicase (FRH) to then damp its own expression through a FRQ-dependent phosphorylation of the WCC, which interferes with its activating role. FRQ is progressively phosphorylated until it can no longer inhibit WCC, and then after getting hyperphosphorylated it is degraded in a proteasome dependent manner^13^. As a result of the negative feedback of FRQ on *frq* expression, both *frq* mRNA and FRQ protein are rhythmically expressed in a daily manner. This cyclical expression is essential for the molecular clock function^11^.

Most of what is known about fungal circadian systems derives from experiments on *N*. *crassa*, but our knowledge on the circadian properties and the underlying molecular and cellular mechanisms of the vast majority of fungi remains extremely limited.

Circadian rhythms (mostly of spore development and liberation as reviewed by Bell-Pedersen *et al*.)^14,15^ were also reported in a handful of other fungal species^16–20^. Circadian phenotypes were described in *Aspergillus nidulans* and *Aspergillus flavus* ^17^, both expressing WC-1 homologs (LreA and LreB) but lacking *frq*. In the case of the phytopathogen *Botrytis cinerea*, a WCC-FRQ based clock has been molecularly confirmed, showing that it regulates daily changes in virulence^19^. Among yeasts, circadian phenotypes were described for the budding yeast *Saccharomyces cerevisiae* ^21^ and *Schizosaccharomices pombe* ^22^. *S*. *cerevisiae* was reported to display circadian oscillations of metabolic parameters and of expression of amino acid and ammonium permeases while in *S*. *pombe*, heat resistance was reported to have an endogenous circadian period of 27 hours^22^.

Interestingly, the ascomycete *Aureobasidium pullulans (Dothideomycetes)* was reported to display daily formation of concentric rings under specific light and culturing conditions^23^ but no circadian basis for such phenotype was found. Although concentric ring formation is a common feature displayed by giant colonies in fungi and other organisms, the mechanisms underlying such morphology vary in different species. In the dimorphic yeast *Schyzosaccaromices japonicum*, light and temperature cycles lead to synchronous cytokinesis causing concentric ring formation^24^. In *Neurospora* the bands are the consequence of rhythmic asexual conidiation, in *A*. *flavus* the bands correspond to rhythmic development of sclerotia (large survival structures) and in other fungi the rings arise because of the presence or absence of aerial hyphae. The interaction between metabolite accumulation and nutrient depletion was proposed as a general mechanism for ring formation in large colonies^25,26^ but the factors determining the timing of the process have not been thoroughly investigated^27^. *A*. *pullulans* displays a wide phenotypic and metabolic plasticity that allows it to be almost ubiquitous in nature and even poly-extremotolerant^28,29^. It is a black yeast-like fungus of considerable biotechnological importance mostly due to the production of pullulan (poly-α-1,6-maltotriose), a polysaccharide currently used for the packaging of food and drugs^30^. It also synthetizes hydrolytic enzymes^31–33^, micosporine-glutaminol-glucoside (UV screen), antimycotic compounds (aureobasidin A)^34^, and some strains are promising biocontrol agents in agriculture^35^. Importantly, it was reported to cause a variety of localized opportunistic infections in humans^36^^,^^37^.

Under the hypothesis that natural circadian traits are more likely to be retained by native “*wild type*” strains rather than their laboratory counterparts, the aim of this work was to identify and characterize the circadian system of an environmental strain of *A*. *pullulans* (CRUB 1823), isolated from the leaf surface of an endemic mountain tree (*Nothofagus pumilio)* in the Patagonian Andes. *A*. *pullulans* represented the most abundant fungi on the surface of the leaves exposed to intense light^38^. We tested *A*. *pullulans* for the presence of spontaneous and self-sustained rhythmic phenotypes, entrainment to light cycles and temperature compensation. We found rhythmic appearance of concentric rings that are entrained by external light/dark cycles and persist under constant darkness in a temperature compensated manner. To further characterize the system, we performed an *in silico* search for the presence of fungal circadian clock gene homologs in the *A*. *pullulans* genome database, and confirmed they were expressed in our Patagonian strain. Among these genes, RT-qPCR experiments demonstrate the light inducibility of *A*. *pullulans* (CRUB 1823) *frq* gene, and the changes in *frq* mRNA levels during a 24 hour time course in constant darkness. While the latter result does not provide yet evidence of *frq* circadian regulation, some parallels can be drawn to the trend that *frq* displays in *Botrytis* and *Neurospora* ^11,16^. *In toto*, the results obtained in the present work suggest the existence of a functional circadian clock in a cold adapted, biotechnology-relevant yeast possessing clock-elements conserved in filamentous fungi.

## Results

### Ring formation evidence for *A. pullulans* endogenous circadian clock

**Figure 1:**
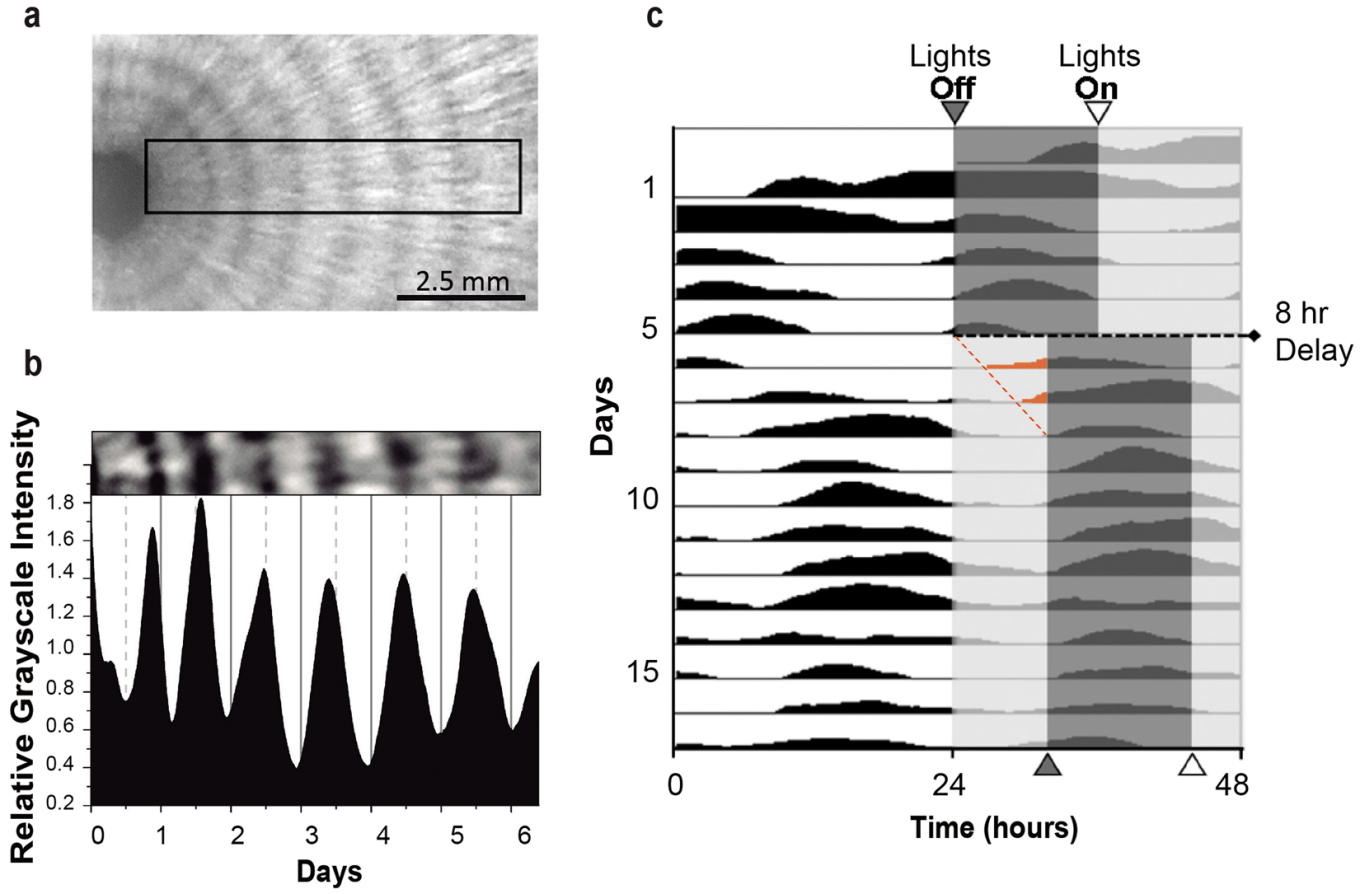
Rhythmic formation of growth rings is entrained to light cycles. (**a)** Representative picture of *A*. *pullulans* grown under light-dark cycles (LD 12 h: 12 h) at 20 °C for 6 days. (**b)** Analysis of the rings observed in the rectangular section marked in (a). The image (top panel) was processed to quantify relative grayscale levels after removing uneven illumination and high frequency noise. (**c)** Representative double plotted actogram of ring formation in *A*. *pullulans* before and after an 8 h delay of the light-dark cycles. Lines in the actogram represent the grayscale levels along consecutive days with each line displaying 48 hours of growth. Light offset and onset are marked with filled and empty triangles respectively at the top and bottom of the actogram. The onset and initial signal for the days after the phase delay are marked in red.

**Figure 2:**
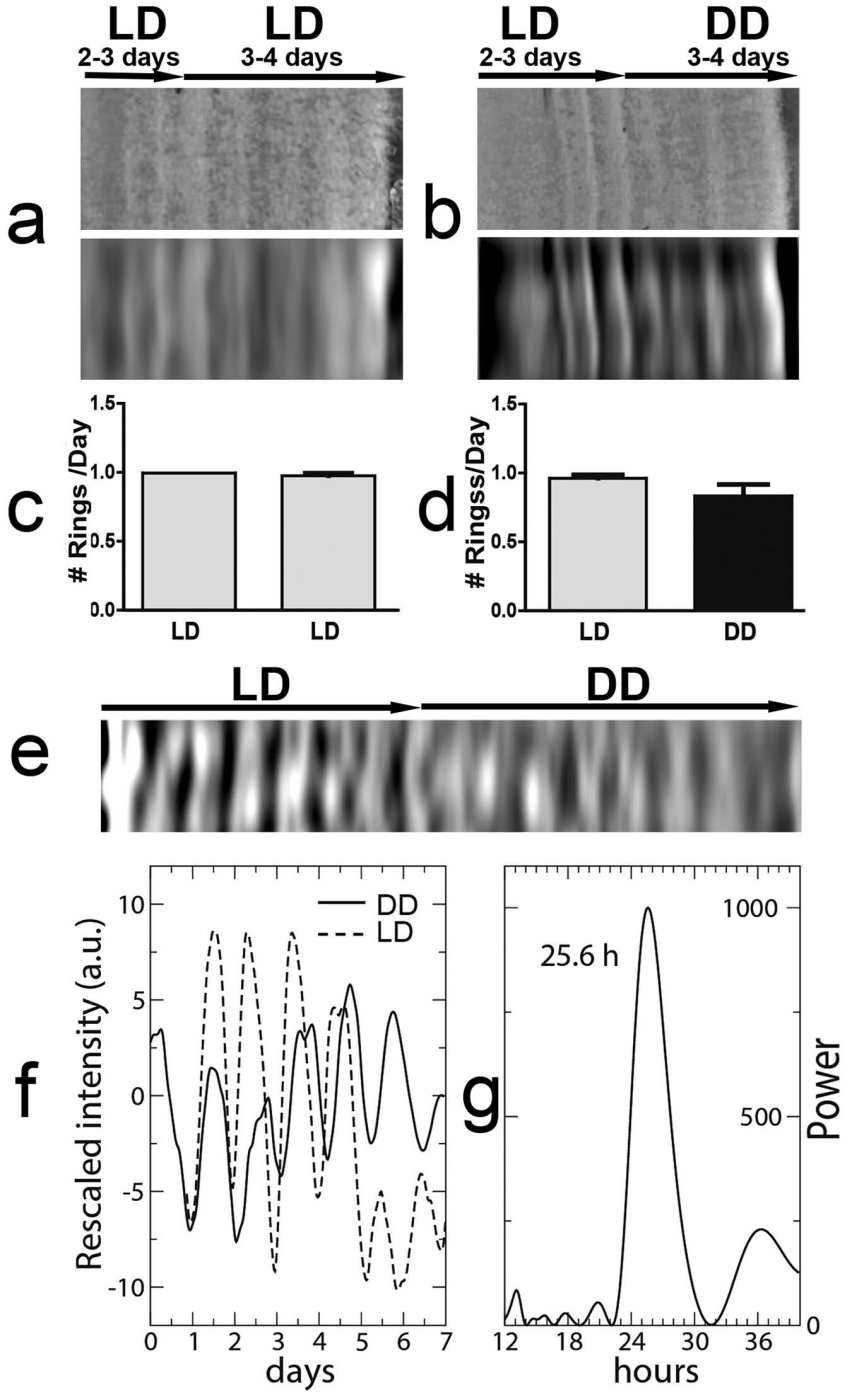
Endogenous circadian rhythm of *A*. *pullulans*. (**a** and **b)** Representative images of *A*. *pullulans* colonies grown under different lighting conditions (LD and DD as represented by the black arrows on top). Each image is shown before (top) and after (bottom) signal processing. (**c** and **d)** Quantification of the number of rings per day obtained from *A*. *pullulans* cultures under each lighting condition. (n = 3 for each condition). (**e)** Representative processed image of an *A*. *pullulans* culture grown under LD and then transferred to DD conditions. (**f)** Quantification of the rings observed in (**e**). The panel represents the relative grayscale intensity profile with the solid line representing DD conditions and the dotted line LD conditions. (**g)** Periodogram for DD, with the peak frequency indicated over the plot.

**Figure 3:**
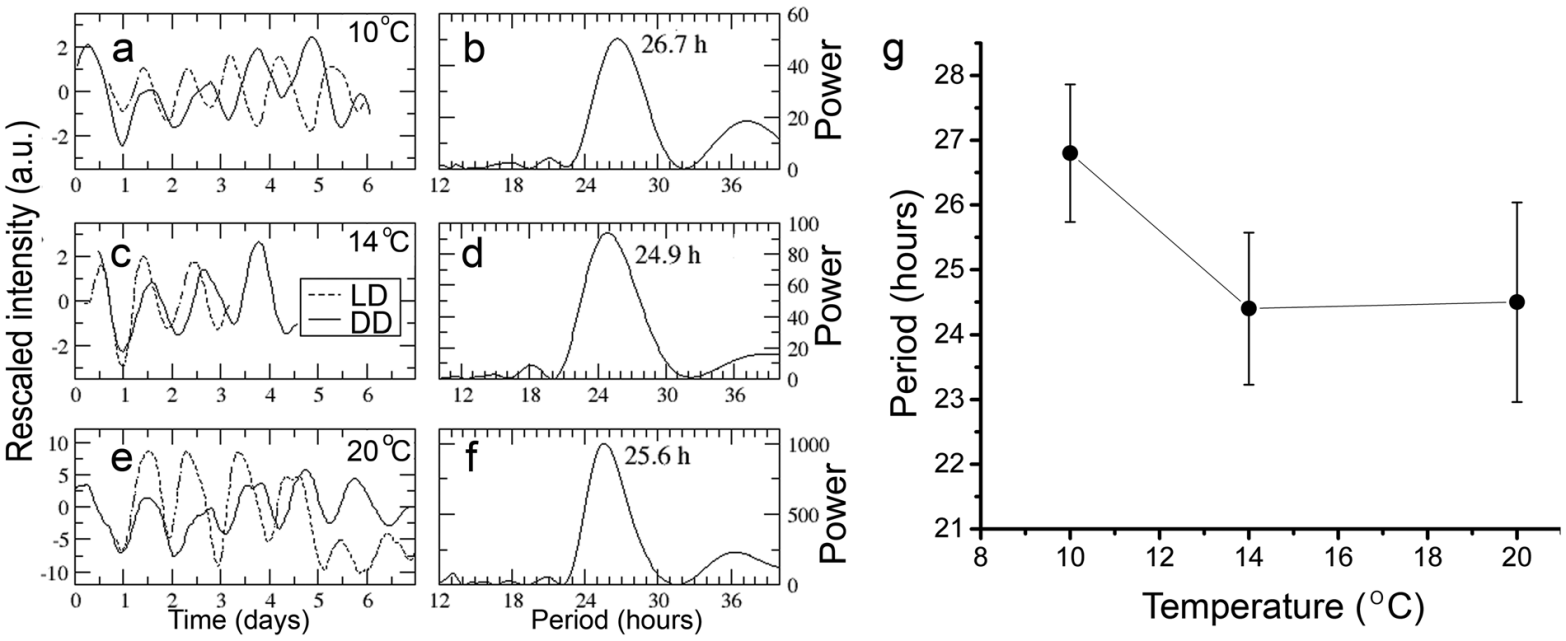
Ring formation in *A*. *pullulans* is temperature compensated. (**a** to **f)** Quantification of the ring formation in three representative cultures of *A*. *pullulans* grown under different temperature and lighting conditions. Panels in the left column show the intensity profile obtained from each culture. Solid lines correspond to the band profile under DD, and dotted lines represent the band profile under LD. Panels on the right are periodograms from each DD intensity profile on the left, and the numbers over the plots correspond to the peak frequency. Each row corresponds to a culture grown at a different temperature: (**a)** and (**b)** correspond to cultures at 10 °C; (**c)** and (**d)** to cultures at 14 °C; (**e)** and (**f)**, to cultures at 20 °C. (**g)** Free-running periods of cultures grown under DD under 10 °C, 14 °C or 20 °C (mean ± SEM, one-way ANOVA, p > 0.05).

Some *A*. *pullulans* strains were reported to display daily morphological changes leading to concentric ring morphology of the colony^23^. To investigate whether the *A*. *pullulans* CRUB 1823 strain can form daily concentric rings, the fungus was grown under light-dark (LD) cycles at 20 °C for over five days. After three to four days under these conditions, the fungus adopted a mycelial morphology displaying a constant radial growth rate (Supplementary Figure S1 and Supplementary Video S1) and forming one concentric ring per day (Fig. 1a,b, Supplementary Figure S2). To further investigate how ring formation is related to external light cycles, four days after the colonies began forming rings the timing of the light cycle was delayed by eight hours (*jet-lag*). After the delay, the ring pattern changed to a longer period for a few days and then stabilized, becoming entrained by the new light cycle (Fig. 1c). To test whether ring formation is generated by an endogenous oscillator or requires the presence of an external light cycle, we investigated if it persists in the absence of external timing cues (constant light and temperature conditions). We found that cultures synchronized to 12:12 h LD cycles could form daily rings even after being transferred to constant darkness (DD) and consistently exhibited the same number of rings as the cultures grown under LD cycles (Fig. 2b,d). The ring pattern at 20 °C exhibited a free-running rhythm with a period of 24.5 ± 1.5 h (mean ± SD) (Figs 2g and 3, see Supplementary Figure S3 for details on signal processing).

A defining property of circadian rhythms is that the free running period remains relatively constant or compensated over a range of physiologically relevant temperatures. *A*. *pullulans* is a widespread fungus that grows under a wide range of different environments such as, phyllosphere, hypersaline waters, rocks, and a wide range of ambient temperatures^39^. *A*. *pullulans* CRUB 1823 strain is native of the Argentinian Northwestern-Patagonia, where the mean temperature is 8 °C and fluctuates between 0 °C and 25 °C across the year. Thus, we tested the temperature compensation properties of the circadian ring formation by growing it at 10 °C, 14 °C and 20 °C. Ring formation was evident at all tested temperatures in both, LD and DD conditions (Fig. 3a,c, and e) and no significant differences in the free-running period (FRP) were observed (26.8 ± 1.2 h, 24.4 ± 1.3 h and 24.5 ± 1.5 h respectively, one-way ANOVA, p > 0.05, Fig. 3g) obtaining a Q_10(10–20 °C)_ = 1.1. These results show that the ring pattern is temperature compensated, a characteristic feature of processes under circadian control. In addition, to our knowledge this is the first evidence of a fungal rhythmic (circadian) phenotype reported at such low temperatures.

Taken together, these results suggest that concentric ring formation in *A*. *pullulans* is a circadian phenotype controlled by an endogenous, temperature compensated oscillator that can be entrained to 24 h light/dark cycles.

### Genes related to circadian clock, sporulation and light responses in *A. pullulans*

**Figure 4:**
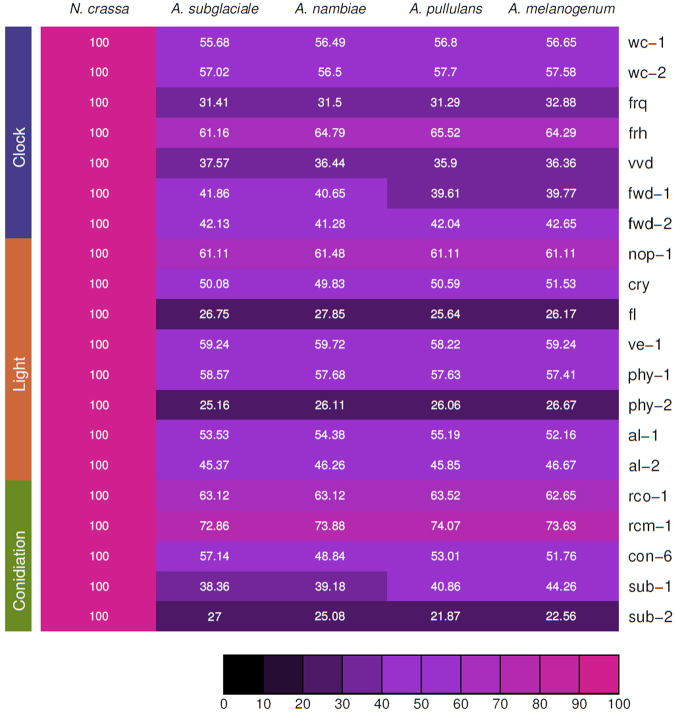
Orthologs of clock, light induced and sporulation genes in *Aurebasidium sps*. (**a)** Percentage of sequence identity to *N*. *crassa* proteins. Clock genes: *white collar-1* (*wc-1*), *white collar-2* (*wc-2*), *frequency* (*frq*), *FRQ-interacting RNA helicase* (*frh*), *vivid* (*vvd*), *F-box/WD-40 domain containing protein-1* (*fwd-1*) and *F-box/WD-40 domain containing protein-2* (*fwd-2*). Light induced genes: *new eukaryotic opsin-1* (*nop-1*), *cryptochrome* (*cry*), *fluffy* (*fl*), *velvetA-like-1* (*ve-1*), *phytochrome-1* (*phy-1*), *phytochrome-2* (*phy-2*), *albino-1* (*al-1*) and *albino-2* (*al-2*). Sporulation genes: *regulator of conidiation-1* (*rco-1*), *regulator of conidiation and morphology-1* (*rcm-1*), *conidiation-6* (*con-6*), *submerged protoperithecia-1* (*sub-1*) and *submerged protoperithecia-2* (*sub-2*).

**Figure 5:**
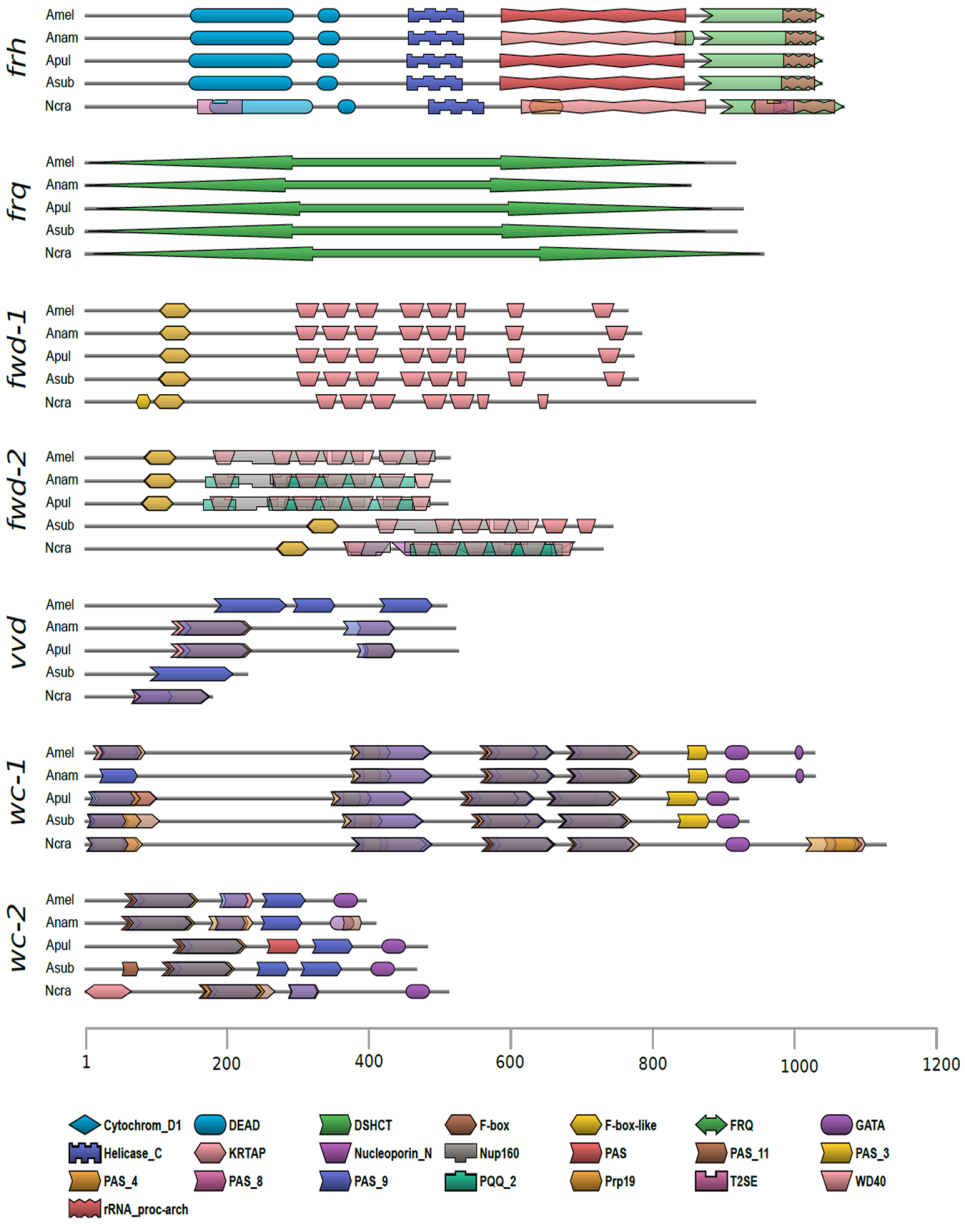
Conservation of the arrangement of functional domains in *N*. *crassa* and *Aurobasidium* core clock proteins. Domain names and accessions from PFAM predicted with HMMER. FRQ-interacting RNA helicase (frh): T2SE (PF00437), DEAD (PF00270), Helicase_C (PF00271), rRNA_proc-arch (PF13234), DSHCT (PF08148) and Prp19 (PF08606). Frequency (frq): FRQ (PF09421). F-box/WD-40 domain containing protein-1 (fwd-1): F-box-like (PF12937), F-box (PF00646) and WD40 (PF00400). F-box/WD-40 domain containing protein-2 (fwd-2): F-box-like (PF12937), F-box (PF00646), Nucleoporin_N (PF08801), WD40 (PF00400), Nup160 (PF11715), PQQ_2 (PF13360) and Cytochrom_D1 (PF02239). Vivid (vvd): PAS (PF00989), PAS_4 (PF08448), PAS_8 (PF13188) and PAS_9 (PF13426). White collar-1 (wc-1): PAS (PF00989), PAS_3 (PF08447), PAS_4 (PF08448), PAS_9 (PF13426), PAS_11 (PF14598) and GATA (PF00320). White collar-2 (wc-2): KRTAP (PF11759), PAS (PF00989), PAS_3 (PF08447), PAS_4 (PF08448), PAS_9 (PF13426), PAS_11 (PF14598) and GATA (PF00320). The bar indicates the number of amino acids.

Recently, it was shown that homologs of *N*. *crassa* circadian clock proteins are present in the predicted proteomes of 64 fungi of the most representative classes^40^, and that FRQ-like sequences are present among early diverging fungi^11,16^. However, in the former study *Aureobasdium* spp. were not analyzed while in the latter the results of the *Dothideomycetes* class were not reported in detail. Under the hypothesis that the components of core circadian oscillators are conserved in fungi we searched for the presence of clock genes in the *Aureobasidium* genus. We performed a bioinformatics analysis aiming at the identification of potential orthologous genes of *N*. *crassa* in four species of *Aureobasidium: A*. *pullulans; A*. *subglaciale; A*. *namibie and A*. *melanogenum*, whose genome had been sequenced recently^29^. We used the predicted coding sequence (CDS) of *Aureobasdium* to search the three related gene sets described in *N*. *crassa*: core clock genes (n = 7), sporulation genes (n = 5) and light induced genes (n = 8)^41^. The candidates were primarily identified by local alignments to *N*. *crassa* genes (Fig. 4). In order to confirm this observation, we investigated the arrangement of functional protein domains. We identified the complete set of predicted homolog proteins known to be core-clock elements, or that can participate in clock modulation (FRQ, WC-1, WC-2, VVD, FRH, FWD-1 and FWD-2), in addition to genes involved in light responses such as *cry*, *ve-1* and *nop-1* as well as sporulation genes (Fig. 5, and supplementary online material http://www.comahue-conicet.gob.ar:8080/c7c4e511cfcf6a4092d99b190649658f/). Moreover, based on RNA-seq evidence from *A*. *pullulans* EXF-150^29^, we confirmed *in silico*, the expression of such genes. All interrogated genes showed at least one related transcript and in most of them (18 out of 21) the sequence overlapped over a 95% of the CDS.

Given all the main components of the circadian core-clock were identified in the genomes of *A*. *pullulans* and related species, we confirmed their expression in the Patagonian CRUB 1823 strain. We selected five representative genes: *wc-1*, *wc-2*, *frq*, *vvd* and *sub-1* and analyzed their expression by RT-PCR. All these genes were found to be expressed in CRUB 1823 (Supplementary Figure S4), confirming the existence of the molecular components of the clock and validating our bioinformatics analysis. Therefore, these findings demonstrate the expression of *N*. *crassa* circadian clock-gene orthologs in *A*. *pullulans (*CRUB 1823).

### Light pulses modulate *frq* expression in *A. pullulans*

**Figure 6:**
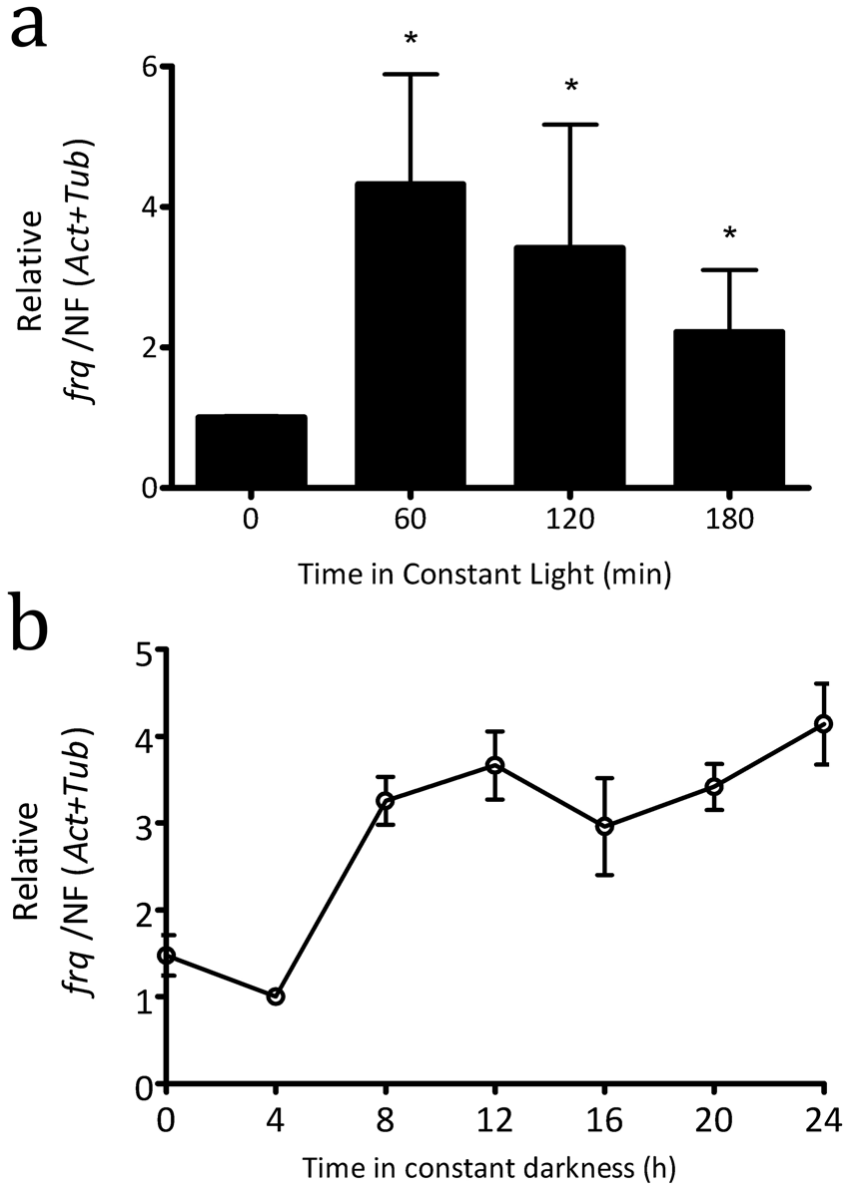
*A*. *pullulans* CRUB 1823 *frq* expression responds to light and varies under constant darkness. (**a)** Analysis of *frq* expression after a light pulse. Gene expression was analyzed by RT-qPCR from samples of *A*. *pullulans* in DD (DD-*frq*) or after a 60, 120 and 180 min. light pulse (LP-*frq*). Values were normalized to DD conditions (control = 1), error bars represent mean values ± SEM, and (*) indicates a significant difference between both groups (Mann–Whitney test, P < 0.05). (**b)** 24 h temporal profile of *frq* expression under DD conditions (mean ± SEM). Cultures were grown for 48 h under LD (12:12 h) cycles and then switched to DD. Culture samples were harvested every 4 h, RNA was then extracted, and *frq* expression was analyzed by RT-qPCR. Values were normalized to DD4 condition (control = 1). *actin* and *tubulin* were used as house-keeping genes.

In *N*. *crassa* light-activated WCC transiently binds to a proximal light response element (pLRE) sequence present in the *frq* promoter acutely inducing its expression^42^. Thus, in order to assess the light control on the transcriptional activity of *frq* in *A*. *pullulans* (CRUB 1823), DD-cultures were exposed to white light for 60, 120 and 180 min and *frq* RNA levels were evaluated by RT-qPCR (Fig. 6a). A significant, 4-fold increase was observed for *frq* transcript levels, 1hr after the light pulse, increment that slowly decreased after prolonged light-stimulation, consistent with a photoadaptation mechanism. Furthermore, we performed a search of consensus LRE motifs based on the model described by He and Liu^43^ on the promoter region of the *frq* ortholog. We found a sequence, 5′-GCATCgagatttgacaggaGCATC-3′, that match the consensus motif which is present upstream of the two estimated transcription start sites (TSS), mapped using RNA-seq evidence, at distances of 425 bp and 584 bp, respectively. These findings demonstrate that the transcription of gene encoding for FRQ in *A*. *pullulans* CRUB 1823 is induced by exposure to white light.

To try to unravel the molecular bases responsible for the process of circadian ring formation the expression of *frq* mRNA in constant conditions was evaluated. Cultures were entrained for 48 hours in LD cycles (12:12 h L:D) and then transferred to DD. Samples of the same culture were harvested every 4 hours, and *frq* mRNA amounts were determined through RT-qPCR (Fig. 6b). A decrease of *frq* levels was observed at the light to dark transition, consistent with what has been described for *Neurospora* and *Botrytis frq* mRNAs. Subsequently, at DD 8, a rapid increase in the levels of *frq mRNA* was detected, to then show a tendency to slightly decrease at DD16, remaining afterwards relatively high up to DD 24. Therefore, while the current data, obtained under the experimental tested condition, do not provide evidence of *frq* circadian oscillations, further work is needed to understand *frq* expression dynamics under extended time courses and different media conditions.

## Discussion

Our study demonstrates the presence of a circadian system in a cold adapted natural isolate of *Aureobasidium pullulans* (CRUB 1823), a yeast-like fungus that bears great biotechnological potential. We observed a concentric ring growth pattern that fulfills all criteria to be considered a circadian phenotype: 1) it is entrained by light cycles, 2) it is present in constant growth conditions with a period close to 24 h and 3) the free running period is temperature compensated (temperature independent) over a wide range of temperatures. Moreover, we confirmed in *A*. *pullulans* the expression of homologs of *frequency*, *white collar-*1 and *white collar-2*, key components of the molecular circadian clock in *N*. *crassa* and the induction of *frq* expression by light pulses.

A significant part of the circadian data generated in *N*. *crassa* has derived from lab strains containing a defined mutation in the *ras-1* gene, which enhances circadian output^44^. Similarly, visualization of circadian banding in WT *Neurospora* isolates requires adding particular chemicals^12,44^. On the other hand, some fungi exhibit vast heterogeneity in terms of light-responses and circadian phenotypes^45,46^ highlighting the fact that monitoring overt circadian phenotypes may not always be straight forward. Nevertheless, the analysis of an *A*. *pullulans* strain (CRUB1823), isolated from a region exhibiting strong environmental changes, allowed clear visualization of such rhythms under standard lab conditions, providing therefore a valuable new fungal model-organism to analyze circadian processes.

We found that the ascomycete *A*. *pullulans* presented a circadian rhythm in ring formation (alternate translucent and opaque bands) when growing on Petri dishes. Similar ring morphologies have been described in fungi and other organisms including bacteria. Although controversial^47^, under particular conditions oscillatory changes in growth rates have been described in *E*. *coli* ^48^. Rhythms have been also reported and commented in other studies involving both *E*. *coli* and *Klebsiella penumoniae* ^49^. Likewise, it has been observed that rhythmic changes in cell densities can lead to ring-like patterns, as part of particular cell-density based regulatory circuits in *E*. *coli*. Ring-like structures, reflecting surface changes and visualized with Congo red or Coomasie have been also observed in *Pseudomona putida* under LD cycles and even up to two days in constant darkness^50^. Yet, besides the evidence of rhythmic oxidation in peroxiredoxins, no molecular data is available on the nature of non-photosynthetic bacterial clocks^51^.

Importantly, phenotypic rhythms in fungi have not been easy to molecularly dissect, other than in *N*. *crassa* and recently in *B*. *cinerea* ^19^. In part this has been because of the lack of clear overt rhythmic phenotypes. While our data provides evidence of a clear rhythmic phenotype in *A*. *pullulans* the exact full nature of these concentric rings still remains to be determined.

Under DD conditions the ring pattern of *A*. *pullulans* is rhythmic for over 6 days and has a FRP at 20 °C of 24.5 h (Fig. 3g). It has generally been assumed that circadian clocks have FRPs close to 24 h in order to maintain a stable phase relationship to the Earth’s 24-h rotational cycle since the inherent cycle cannot be too far away from the environmentally driven cycle for optimal performance^52^. Therefore, although the FRP observed in *A*. *pullulans* is longer than 24 h, it lies within the expected range for a *circa*-day rhythm controlled by the clock. In fact, FRPs in fungi cover a broad range with the FRP of *N*. *crassa* being shorter than 24 h (22.5 h)^11^ and the rhythm of sclerotia formation in *A*. *flavus* having an FRP of 33 h^17^ one of the longest natural occurring circadian rhythms. Such large variations in the FRPs could arise from differences in the molecular mechanisms underlying circadian rhythm generation underscoring the importance of studying circadian systems in other fungal species.

Temperature compensation is a critical feature of circadian pacemakers since the system has to be able to entrain to 24 h cycles despite large variations in ambient temperatures (i.e. daily, seasonal and latitudinal temperature changes). Temperature cycles can entrain circadian pacemakers, but under constant conditions the FRP should be similar over a broad temperature range. Because *A*. *pullulans* is a widespread fungus that can easily colonize different environments^39^ under a broad range of temperatures, we tested temperature compensation of ring formation between 10 °C and 20 °C, a range covering the average temperatures of the area where the CRUB 1823 was isolated from. We found no significant differences in the FRP of the ring pattern in cultures maintained at 10 °C, 14 °C or 20 °C (26.8 ± 1.2 h, 24.4 ± 1.3 h and 24.5 ± 1.5 h respectively, Q_10(10–20 °C)_ = 1.1, Fig. 3). Interestingly, our data also shows for the first time in fungi a rhythmic phenotype with an easy read-out even at temperatures as low as 10 °C, making *A*. *pullulans* a new and very interesting model-organism to study the molecular mechanisms responsible for temperature compensation in circadian clocks.

The extent to which circadian rhythms and their underlying genetic components are conserved among fungi is unknown. However, in the past years several clock components were identified in different fungal genome databases and these data show that while WCC appears to be relatively well conserved, FRQ was lost several times during fungal evolution^11,40^. One example of this divergence is observed in the class *Dothideomycetes*, where it was reported that the pathogenic ascomycete *Cercospora kikuchii* displays a circadian rhythm of hyphal melanization^18^. Interestingly, the authors were able to detect *wc-1* and *wc-2*, but they could not identify any *frq* homolog. In contrast, we identified *wc-1*, *wc-2* and *frq* genes and moreover, we were able to measure an increment in *frq* mRNA levels after light stimulation. *In silico* analysis of *Mycosphaerella graminicola*, another representative of the *Dothideomycetes*, also reveals the presence of FRQ-like sequences^11^. This suggests that FRQ may not be a conserved molecular clock component even among species of the same class, while other components such as WC-1 and WC-2, which are determinant in fungal light sensing and also participate in other non-circadian processes are conserved across fungi^11,53^. As more fungal genomes are sequenced and circadian phenotypes are found in fungi, a better understanding will emerge regarding the extent of conservation of molecular clock components and their evolution.

Photoperception is a key process that allows organisms to be susceptible to light cues and thus, properly respond to changes in the environment. We are currently studying the conserved components of fungal light perception in *A*. *pullulans*. In *N*. *crassa* WC-1, a member of the GATA family of transcription factors, is also a blue light receptor capable of sensing and mediating responses to light^11^. WC-2 is its partner in the light signaling pathway and both are required for all light responses in addition of their roles in the circadian feedback loop^11^. Another blue light photoreceptor is the VIVID protein, which is dependent on the WCC and plays important roles in regulating light responses and photoentrainment of the clock in *N*. *crassa*. As shown here *A*. *pullulans* encodes for orthologs of the transcription factors WC-1, WC-2 and a VIVID-like protein, which exhibit characteristic key conserved domains (Figs 4 and 5). *A*. *pullulans* responds to white light at the transcriptional level as seen by the increase in expression levels for *frq* gene after a light pulse, effect that decreases after long exposure to light, consistent with a photoadaptation mechanism (Fig. 6a). Future work will assess the role of LRE sequences found in *frq* promoter in mediating light responses and the response of other photoreceptors and transcription factors to light pulses.

The functional role of the circadian system and the selective advantage it could confer to *A*. *pullulans* is currently hard to assess, but further studies of the banding pattern and its molecular basis combined with experimental designs addressing interaction with different substrates and environmental conditions will increase our knowledge about its evolutionary relevance. It was reported that *A*. *pullulans* is able to form hyphae with chlamydospores containing melanin in their cell walls^23^, and Bluhm *et al*., described that hyphal melanization in *C*. *kikuchii*, (of the same class as *A*. *pullulans*) is under circadian control^18^. So, if the circadian clock is regulating melanization this could lead to an increase in the survival rates of the fungus as melanin decreases sensitivity to UV irradiation and regulates processes involved in pathogenesis. The circadian clock could also modulate the production of other compounds that confer the fungus an ability to survive in different ecological niches. Further efforts will focus on identifying the mechanisms underlying band formation and their relationship with morphological changes and melanin production. Recent studies have highlighted the role of circadian clocks as an organizer of metabolism in fungal cells^54^. Therefore, it is plausible to predict that some metabolites, including proteins or compounds of biotechnological or industrial value, may oscillate daily. Exploring if such rhythms exist, and understanding how they are regulated is the first step in order to optimize growth conditions and products yield. Elucidating the molecular basis of any of such putative regulatory processes would allow the design of potentially hyperproducing mutants through genetic engineering strategies

It is interesting that the molecular dissection of fungal circadian systems, other than the one in *N*. *crassa*, has started to reveal intriguing insights into the complexity and diversity of such systems. Thus, the study of the *Botrytis cinerea* clock has shown that genetic inactivation of the WCC in this phytopathogenic fungus does not completely abrogate molecular and phenotypical responses to light^55^, something that contrasts to the pivotal role that WCC has in *N*. *crassa* in light-signaling, although it is otherwise closer to what has been seen in other photobiology models^53^. Moreover, the rings that are observed under LD cycles in *B*. *cinerea* (and which do not persist under DD), are strengthened in WC-1 mutants^55^. In addition, the data has confirmed that the *B*. *cinerea frq* homologue (*bcfrql1*) is a core-clock component and key for allowing increased virulence at night-time versus day-time^19^. Surprisingly, on top of this central circadian role, *bcfrql1* appears to regulate other aspects of *B*. *cinerea* biology even in conditions where clock function is not relevant (such as LL), confirming unexpected extra-circadian roles for a FRQ protein.

On the other hand, results from *Pyronema confluens* confirmed temperature compensated rhythmic expression of a *frq* homolog^20^. Nevertheless, such expression was not entrained by light and did not produce any overt circadian phenotype.

Therefore, the future molecular characterization of rhythms in *A*. *pullulans*, an organism with a robust circadian phenotype, will provide interesting and informative insights into the conservation and evolution of circadian mechanisms. Particularly, the fact that rhythms are readily visible at temperatures as low at 10 °C, and that this fungus can be found in diverse environments, may also provide new ways to approach the fascinating and yet rather unknown mechanisms underlying temperature compensation.

While the preliminary data on *A*. *pullulans frq* expression reveal a clear decrease from a light to dark transfer followed by fluctuations under constant darkness, the evidence is insufficient yet to call its expression circadian. Importantly, in our own experience with *N*. *crassa* we have observed that media composition and the way time courses are performed (i.e solid vs liquid media) can have an important effect in the strength and quality of the molecular rhythms, assayed as RT-qPCR or Western blot^13,56^.

In summary, our data demonstrate the presence of a functional circadian oscillator in *A*. *pullulans*, paving the road to future molecular studies in a biotechnologically and environmentally interesting new fungal circadian model.

## Methods

### *Aureobasidium pullulans* strains

A strain of *Aureobasidium pullulans* CRUB 1823 was isolated from the leaves of the tree *Nothofagus pumilio* present in Otto hill in the North-Western Patagonia region. This hill is located on the southern side of Lake Nahuel Huapi, in the city of San Carlos de Bariloche, Río Negro. The strain was identified by sequencing the regions D1/D2 of 26 S rDNA. The strain is deposited in the Yeast Culture Collection of the IPATEC (Bariloche, Argentina) and it is available upon request through MTA (contacto.ipatec@com ahue-conicet.gob.ar). The genome sequences of four varieties of the genus *Aureobasidium pullulans* (*A*. *pullulans; A*. *subglaciale; A*. *namibie* and *A*. *melanogenum*) were published^29^.

### Culture conditions

*A*. *pullulans* strain CRUB 1823 was grown at 20 °C in Petri dishes containing GSA media (0.2% glucose, 0.2% soy peptone and 1.5% agar). Petri dish cultures were grown in 24-h light-dark cycles (12:12 LD) for 3 to 7 days prior to transfer to constant darkness (DD) in controlled environmental incubators equipped with cool white light fluorescent tubes (light intensity 60 micromoles/m2/s; wavelength 400–720 nm). When cultures were grown in LD and then transferred to DD, a red led light was used to mark the transition to DD.

For light-pulse and time course experiments liquid cultures were performed by growing *A*. *pullulans* in 1000 ml flasks containing 400 ml of YM media (0.3% yeast extract, 0.3% malt extract, 1% glucose and 0.5% peptone), at 20 °C with constant agitation (125 rpm). After inoculation *A*. *pullulans* was grown for one day in a 12:12 LD cycle, and then transferred to constant darkness for exactly 24 hour prior to exposing cultures to white light (intensity of 60 micromoles/m2/s; wavelength 400–720 nm) for 60, 120 and 180 min and then samples (20 ml) were collected. For time course experiments the cultures were grown in LD (12:12 h) conditions for 48 h prior transfer to constant darkness at 20 °C, maintaining constant agitation (125 rpm). After this, every 4 h 20 ml were harvested in a temperature-controlled darkroom equipped with low-intensity red-safety lights, and samples were immediately frozen in liquid nitrogen. Experiments were performed in triplicate.

### Temperature compensation experiments

*A*. *pullulans* was grown on GSA media at different temperatures: 20 °C, 14 °C or 10 °C. At each temperature, plates were incubated in a 12:12 LD cycle for 3 to 7 days and then transferred to DD culture conditions for another 6 or 7 days. Each plate was marked in the LD to DD transition, as a reference of time.

### Analysis of daily and circadian rhythms

By means of time lapse video recordings of growing cultures we determined that growth rate is constant for many days for each culture. Therefore cultures were not disturbed while growing in constant conditions. By the end of the experiments or at the time of changes in light conditions, cultures were photographed with a digital camera and images analyzed by counting the number rings formed and assessing changes in the grayscale intensity of the concentric rings. The time scale was obtained by dividing the distance (in pixels) between the last and the first rings by the number of hours elapsed from the formation of the first ring to the formation of the last ring.

From the image we extracted a rectangle where bands were approximately parallel to the shorter side of the rectangle (i.e. the rectangle extends radially from the seed to the border of the fungus). The images were smoothed with a Gaussian blur filter (sigma = 3) to remove high frequency noise and a pseudo flat-field illumination correction filter was applied to correct for uneven illumination. Then, we averaged the intensity of each row of pixels (i.e. pixels with the same “radial” coordinate), obtaining a curve of intensity as a function of the position of the row (Fig. 1a,b). Image analysis was performed with ImageJ software (http://rsbweb.nih.gov/ij/) and the ActogramJ plugin^57^ was used to generate the actograms (Fig. 1c).

To extract the daily component in the grayscale changes which reveals the presence of bands, we performed a detrending of the curve by subtracting the best quadratic function fit. The period of the resulting curves was analyzed by performing a Discrete Fourier Transform. All operations were performed using mathematical software (Mathematica).

### RNA extraction and Real-time quantitative RT-PCR (RT-qPCR)

All samples were kept at −80 °C until further purification. Total RNA was isolated using TrIzol reagent (Invitrogen) as previously described^58^. Total RNA quantity and quality was verified using NanoDrop (Thermo Scientific) and by electrophoresis in an agarose gel (1% w/v). RNA was further purified using the RQ1 RNasefree DNase (Promega), following the manufacturer’s instructions. Absence of genomic DNA contaminations in the samples was confirmed by RT-minus reactions. Thereafter, RNA samples (1 µg) were reverse transcribed using the MMLV reverse transcriptase (Promega), according to manufacturer’s directions. One µl of cDNA was used in each RT-qPCR reaction.

Transcript quantification was achieved using the SensiMixPlus SYBR Green kit (12.5 ml reactions; Quantace) and the StepOnePlus™ Real-Time PCR System (life technologies) as described in manufacturers’ manuals. The RT-qPCR was performed as follows: 10 min at 95 °C followed by 40 cycles of 15 s at 95 °C, 15 s at 58 °C and 15 s at 72 °C, followed by a melting cycle from 55 to 95 °C to check for amplification specificity. Cq values were acquired during the annealing period of the RT-qPCR. These values were used to obtain the accurate ratio between the gene of interest (GOI) and the expression of the reference gene employed for normalization, using the formula previously described^59^.

The primers utilized were: FRQ-Fw, 5′-CACCAAGAGTTGCCACCTTC-3′, FRQ-Rv, 5′-TGCTCAAATACGGCATGTCG-3′; ACTIN-Fw, 5′-TGTACGGCAACATCGTCATG-3′′, ACTIN-Rv, 5′-TTCATGGATGAGGGAGCAAGAG-3′. TUB-Fw, 5′ TGCATGCTTTCCAACACCAC-3′, TUB-TV, 5′AAGCGCGCTTTGAGTACATG-3′

### Bioinformatics methods

Genome sequences and predictions were downloaded from the Joint Genome Institute portal (JGI)^60^. The five datasets downloaded corresponded to *Aureobasidium pullulans var*. *subglaciale* EXF-2481, *Aureobasidium pullulans var*. *namibiae* v1.0, *Aureobasidium pullulans var*. *melanogenum* v1.0, *Aureobasidium pullulans var*. *pullulans* EXF-150 v1.0, assembled by Gostinčar *et al*. (2014)^29^ and *Neurospora crassa* OR74A v2.0. The ortholog genes from predicted proteomes of four strains were primary identified with TBLASTN (e-value < 1e-5)^61^ using *N*. *crassa* sequences. Ortholog sequence alignments and domain mappings HAMMER^62^ and PFAM database^63^ were manually compared to functional domains annotated in UNIPROT (UniProt Consortium, 2011). Two sets of processed RNA-seq reads for *A*. *pullulans var*. *pullulans* (EXF-150) were downloaded from JGI (Project: 1013365). Transcriptomes *de-novo* were inferred with rnaSpades (SPAdes v3.9)^64^. Alignments to predicted CDSs were performed with Blat (-minScore = 100)^65^ and their sequence coverage were retrieved.

## Electronic supplementary material

Supplementary Information

Supplementary Video S1
