# Supplementary material for: Spontaneous circadian rhythms in a cold-adapted natural isolate of Aureobasidium pullulans
Source: Sci Rep. 2017 Oct 23;7:13837. doi: 10.1038/s41598-017-14085-6 (PMC5653790; doi:10.1038/s41598-017-14085-6)

Spontaneous Circadian Rhythms in a Cold-Adapted natural isolate of *Aureobasidium pullulans*.

Diana L. Franco, Paulo Canessa, Nicolás Bellora , Sebastián Risau-Gusman, Consuelo Olivares-Yañez, Rodrigo Pérez-Lara, Diego Libkind, Luis F. Larrondo, and Luciano Marpegan

Supplementary online material:

Interactive table and database containing data from the bioinformatics analysis performed in the current work can be found at:

http://www.comahue-conicet.gob.ar:8080/c7c4e511cfcf6a4092d99b190649658f/

Supplementary Video S1:

Representative time-lapse recording of an A. pullulans colony growing over 7 days. Images were obtained every 3 minutes using a Noganet NGW-091 high definition webcam controlled with Timelapse software (TNL enterprises) and processed with ImageJ software for flat-field correction, noise removal and frame stacking. After image processing, each frame in the video corresponds to a one hour interval. Light/dark cycles were generated using red, green, blue and ultraviolet light emitting diodes during the light phase and only red LEDs during the dark phase. Oblique Illumination was set to increase detection of the leading edge and a red filter (#027, medium red from Lee Filters, USA) was utilised to avoid image fluctuations between light and dark phases of the LD cycles.

Supplementary Figure legends:

Supplementary Figure S1. Radial growth of *A. pullulans* colonies is constant and independent from lighting conditions. (a) Radial growth rate of colonies kept under LD or DD conditions for 72 hours showed no significant differences (n=4 for each group). **(b)** Average kymograph (x position vs. time plot) of a time lapse recording of an *A. pullulans* colony growing for 10 days.

Dark areas at the top correspond to the background and the white areas at the bottom correspond to the colony. The edge between those areas correspond to the leading edge of the mycelium. Radial growth is evidenced by the white signal increasing linearly towards the right side of the image. **(c)** Plot of the leading edge of the colony and linear fit (dashed red line) showing that radial growth rate (slope of the linear fit) can be considered constant for at least 7 days.

Supplementary Figure S2. Rhythmic ring formation in *A. pullulans*. (a) Image of a section of an *A. pullulans* colony grown under LD conditions showing the typical growth with alternating opaque and translucent bands (marked with black and white triangles, respectively) forming the concentric rings (scale bar: 0.25 mm) **(b)** Higher magnification image of the area marked in (a). A large number of spore clusters around the hyphae can be observed (scale bar: 0.1 mm). **(c)** High magnification image of a clump of spores similar to the ones observed in (b) (scale bar: 0.1 mm). **(d)** Section of a Sudan Black stained *A. pullulans* (scale bar: 0.1 mm).

Suppplñementary Figure S3. Signal processing for quantification of rhythmic ring formation in *A. pullulans* colonies. Images of *A. pullulans* require filtering of low and high frequency signals in order to analyze fluctuations in the circadian range. Panels (a) to (g) describe the operations that were performed on the original signals in order to find the endogenous period for each culture. **(a)** Image of half of a colony of a representative culture after the first detrending. Areas with no markings or evidence of zonification were selected for further processing. **(b)** Greyscale intensity was measured in the selected area of the original image (green trace) and then divided by the low frequency trend (dashed red line) and multiplied by the average signal. **(c)** Detrended data was then spatially filtered to remove horizontal components in the images. Filtering was performed transforming the image to the frequency domain through fast Fourier transform (FFT), generating a mask with a horizontal window and then transforming the image back to the spatial domain through an inverse FFT. **(d)** The data was then smoothed (3x3 gaussian blur, blue trace) and the remaining low frequency components (dashed red trace) were removed to obtain the final processed data observed in panels **(e** and **f)**. **(g)** Plot of the

result of a Lomb-Scargle periodogram with the peak corresponding to the estimated period of the signal.

Supplementary Figure S4. *A. pullulans* CRUB 1823 express clock genes and respond to light at transcriptional level. (a) Representative RNA electrophoretic run (1% agarose gel) stained with ethidium bromide to verify RNA integrity of the DD and LP samples. 5μg of RNA were loaded in each lane. **(b)** Representative RT-PCR clock-gene expression analysis from total RNA obtained from *A. pullulans* cultures grown in liquid medium under DD, or after a 60 min light pulse. Since no previous works studying clock-gene expression in *A. pullulans* were reported, two sets of primers were used to increase the possibility of obtaining positive results. Lanes 2-5 with set (a) and lanes 6-15 with set (b). Genes evaluated in each lane were: 2 and 6, *frq*; 3 and 7, *wc1*; 4 and 12, *actin*; 5 and 13, *tubulin*; 8-*wc2*; 9-*vvd*-like; 10-*sub1*; 11-*sub1* after a light pulse (LP). RT-PCR control reactions without cDNA template (lane 14, NTC) and with no reverse transcription (lane 15, RT(-)) were performed to rule out genomic DNA or other contaminations in the sample.

Set (a): *frq*-Fw, 5′-CACCAAGAGTTGCCACCTTC-3′, *frq*-Rv, 5′-TGCTCAAATACGGCATGTCG -3′; *wc-1*-Fw, 5′-TCACCATCAGCAGTCAAGGT -3′, *wc-1*-Rv, 5′- CTGCTCAGTGTGAAGCGATC-3′; *actin*-Fw, 5′-ACTGGGACGACATGGAGAAG-3′, *actin*-Rv, 5′-GGATGGAGACGTAGAAGGC-3′; *tubulin*-Fw, 5′-CTGGGAGCTCTACTGTCTCG -3′, *tubulin*-Rv, 5′- CCTTGCCAGTGATCATCTGC -3′.

Set (b): *frq*-Fw, 5′- AGCAGCAAACGCCTTTTACG-3′, *frq*-Rv, 5′- CCACATCCATGAAACAGGAAGC -3′; *wc*-1-Fw, 5′- TGTGCCAATTGCCATACCAG -3′
wc-1-Rv, 5′- TTGCAAAGGTCGCGATTTCC -3′; *wc*-2-Fw, 5′- GACGCCAAATTACCAGGGAATG -3′, *wc*-2-Rv, 5′- AATGCGCTGTCCAGGTTTTG -3′; *vvd*-Fw, 5′- TCAGCATTGCGCACTATTCG -3′,
vvd-Rv, 5′- TTCAGATGTGCCATGCGTTC -3′; *sub*-1-Fw, 5′- AGGCGCAAGAACAGCAATTC -3′,
sub-Rv, 5′- CGCCAGCTTTTGACTCTTCAG -3′

Supplementary Figure S1.

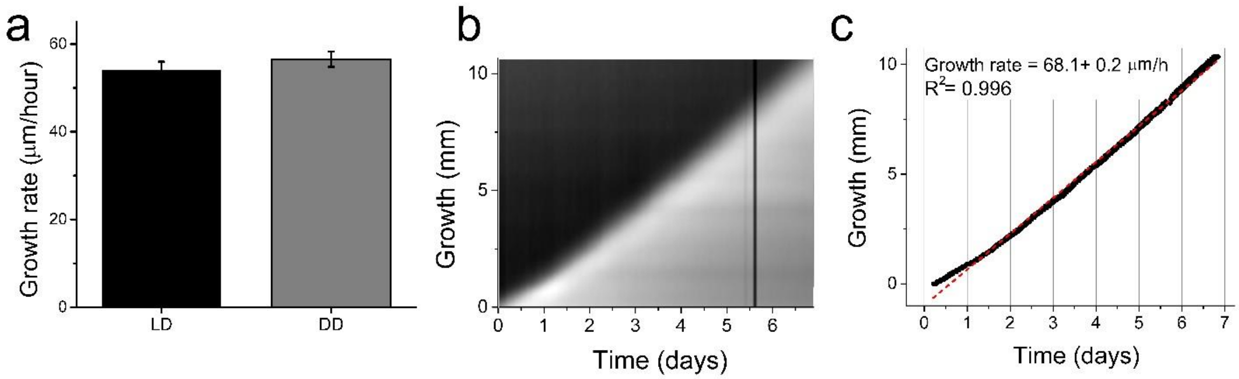

Supplementary Figure S2.

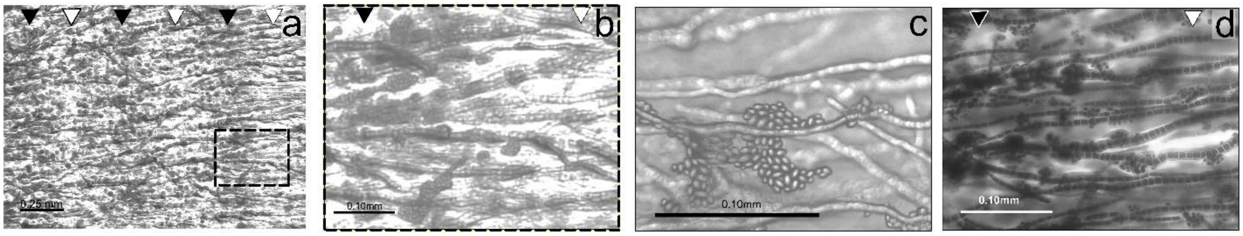

Supplementary Figure S3.

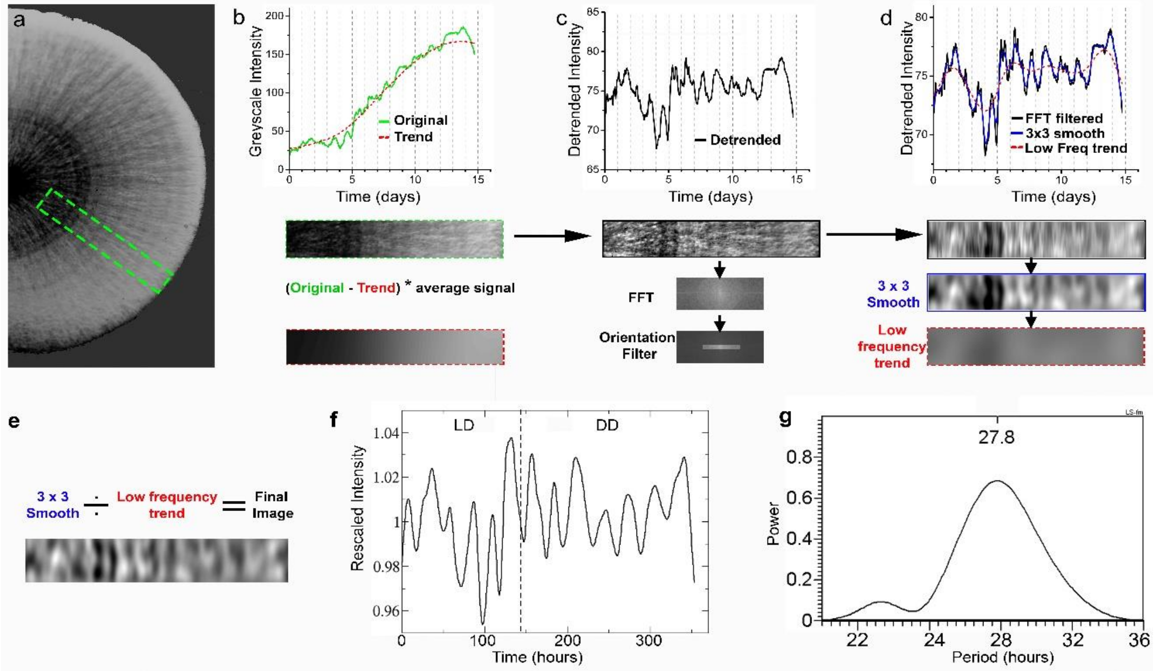

Supplementary Figure S4.

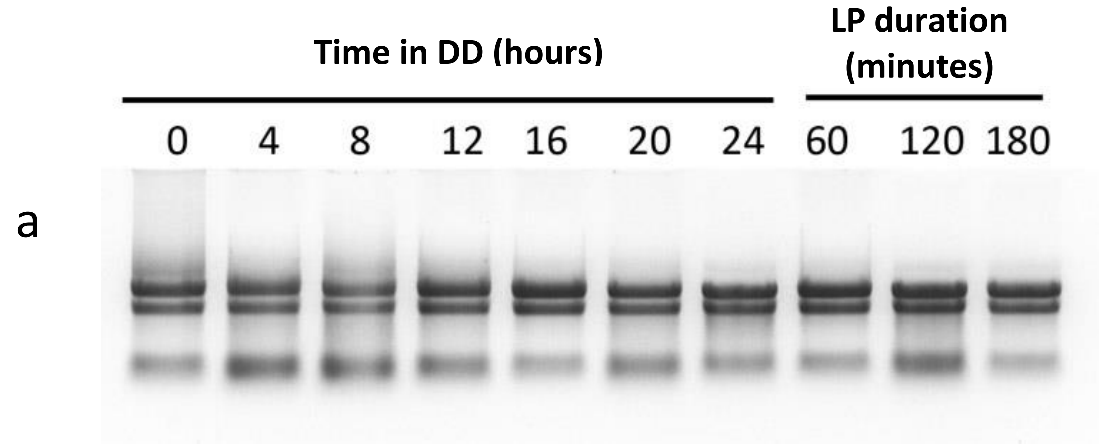

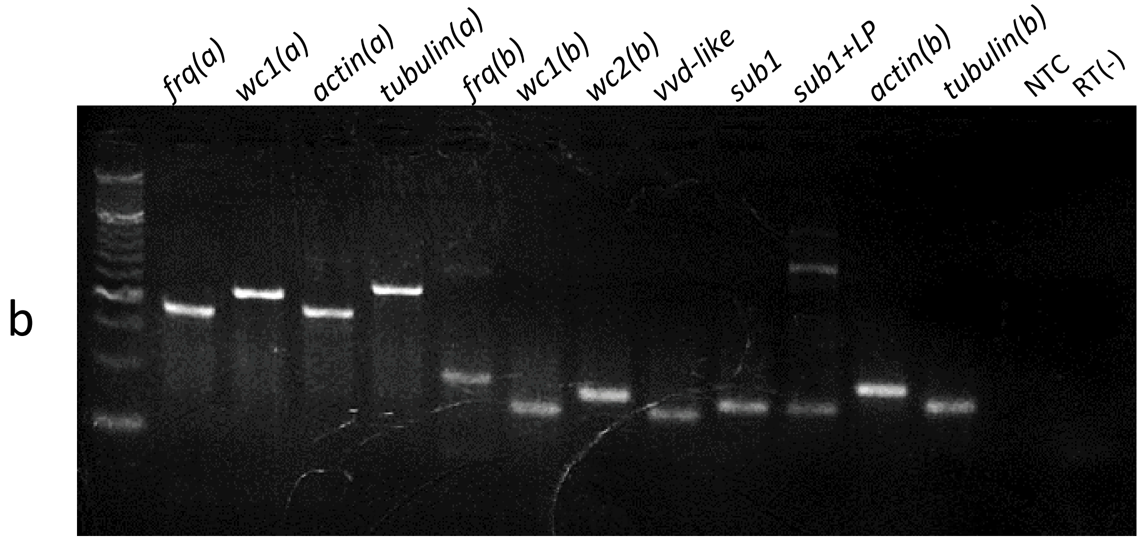

Supplement: Supplementary file 1 — Supplementary Information [file 41598_2017_14085_MOESM1_ESM.pdf]
