# Supplementary figures and images for: Spontaneous circadian rhythms in a cold-adapted natural isolate of Aureobasidium pullulans
Source: Sci Rep. 2017 Oct 23;7:13837. doi: 10.1038/s41598-017-14085-6 (PMC5653790; doi:10.1038/s41598-017-14085-6)

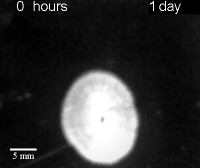

Supplement: Supplementary file 2 — Supplementary Video S1 [file 41598_2017_14085_MOESM2_ESM.gif]
